# The Evolution of Prosocial and Antisocial Competitive Behavior and the Emergence of Prosocial and Antisocial Leadership Styles

**DOI:** 10.3389/fpsyg.2019.00610

**Published:** 2019-06-25

**Authors:** Paul Gilbert, Jaskaran Basran

**Affiliations:** Centre for Compassion Research and Training, College of Health and Social Care Research Centre, University of Derby, Derby, United Kingdom

**Keywords:** antisocial, compassion, competitive behavior, leadership, prosocial

## Abstract

Evolutionary analysis focuses on how genes build organisms with different strategies for engaging and solving life’s challenges of survival and reproduction. One of those challenges is competing with conspecifics for limited resources including reproductive opportunities. This article suggests that there is now good evidence for considering two dimensions of social competition. The first, has been labeled as *antisocial* strategies, to the extent that they tend to be self-focused, threat sensitive and aggressive, and use tactics of bulling, threatening, and intimidating subordinates, or even injuring/killing competitors. Such strategies can inhibit care and affiliative social interactions and motivation. The social signals emitted stimulate threat processing in recipients and can create stressed and highly stratified groups with a range of detrimental psychological and physiological effects. Second, in contrast, *prosocial* strategies seek to create relaxed and secure social interactions that enable sharing, cooperative, mutually supportive and beneficial relationships. The friendly and low/no threat social signals emitted in friendly cooperative and affiliative relationships stimulate physiological systems (e.g., oxytocin, the vagus nerve of the parasympathetic system) that downregulates threat processing, enhances the immune system, and facilitates frontal cortical processes and general wellbeing. This article reviews the literature pertaining to the evidence for these two dimensions of social engagement.

All life forms face life tasks of having to acquire resources and defend themselves from threats in the pursuit of survival and reproduction. Some of the strategies life forms’ use are more successful than others (Davies et al., 2012). Some require ways to interact with conspecifics who are pursuing the same life tasks and resources and can pose opportunities or threats. Thus, evolution is underpinned by competitive behavior in the pursuit of survival and reproduction (Buss, 2015). The strategies for securing resources vary. Both within and between species, competition can be fierce and combative where the strongest wins, but they can also involve degrees of altruism (Warneken and Tomasello, 2009; Ricard, 2015) and the creation of supportive, trusting, mutually cooperative and reciprocal, and affiliative relationships (Penner et al., 2005; Gilbert, 2015, 2017a,b; Dunbar, 2016; Seppälä et al., 2017). These “friendly” and altruistic strategies turn out to provide advantage in securing resources and reproductive opportunities by rendering conspecifics attractive to each other, enabling mutually advantageous relating in roles such as breeding, offspring caring, and cooperative alliance building (Hardy and Van Vugt, 2006; Phillips et al., 2008). However, they also arise from competition to be attractive to and chosen by audiences (Barkow, 1989; Gilbert, 1989/2016, Gilbert et al., 1995; Etcoff, 1999; Sznycer et al., 2016). Conspecific interactions along dimensions of hostile-threatening versus friendly-helpful have major impacts on the physiological regulation of participants (Colonnello et al., 2017), including epigenetic influence (Conway and Slavich, 2017). These dimensions of (competitive) interpersonal interaction have been frequently referred to in various ways, such as “antisocial” and “prosocial” strategies respectively (Cheng et al., 2014; Brañas-Garza et al., 2016; Gilbert, 2018).

This article will outline some of the evolutionary and social contextual thinking behind these dimensions. Specifically, it will explore how different forms of competition are reflected in specific antisocial versus prosocial competitive motives, emotions, and behavioral strategies and are linked to personality and leadership styles. We also consider how social contexts recruit variations in these strategies, enabling an insight into how antisocial and prosocial leadership styles can emerge within communities.

## The Evolution of Life Tasks

**Figure 1:**
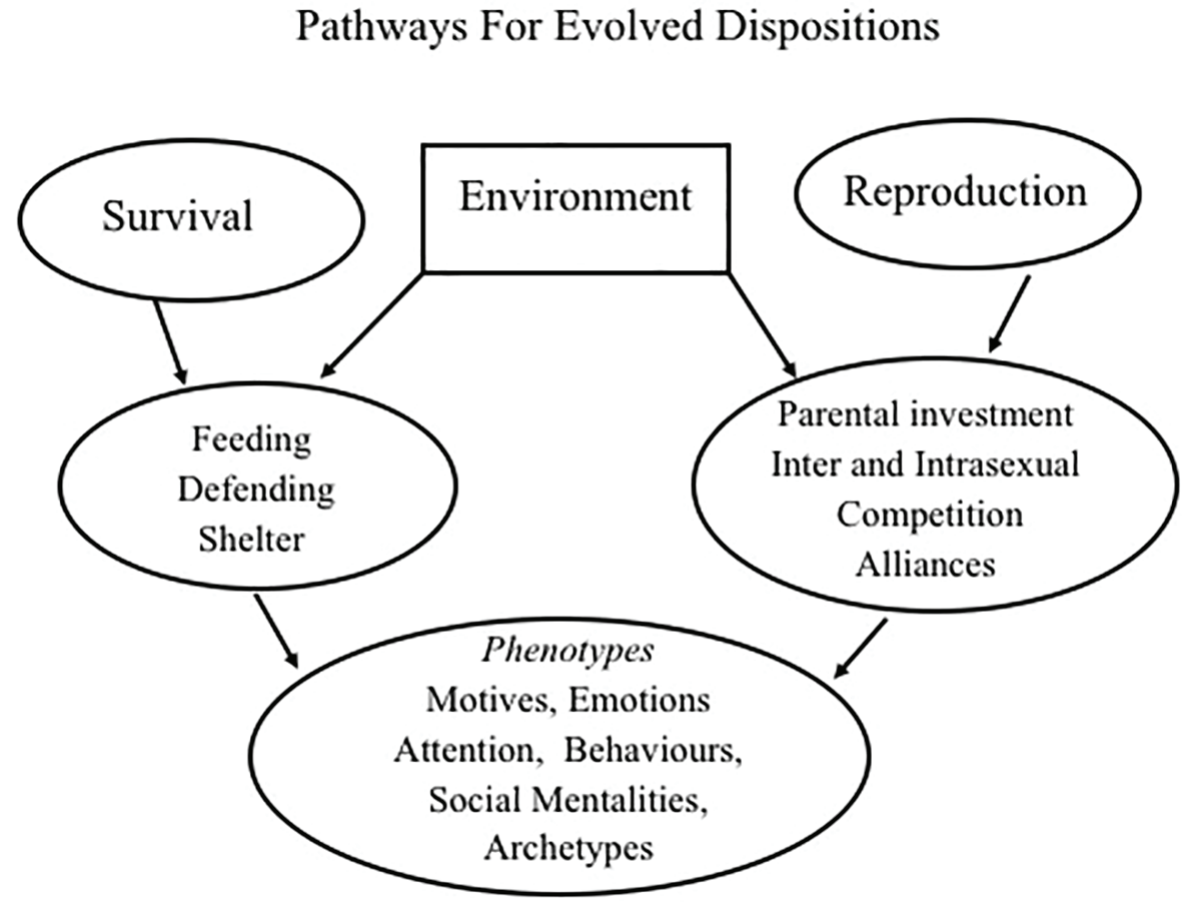
Relationship between evolved strategies, phenotypes, motives, and social mentalities. From Gilbert (2018) Living like Crazy with kind permission from Annwyn House Publishers.

To begin the journey then, and as noted, the two main life tasks of all living beings are survival and reproduction. These give rise to a variety of strategies and phenotypes for social relating that many evolutionary psychologists have identified and are depicted in Figure 1 (Gilbert, 1989/2016; Barrett et al., 2002; Davies et al., 2012; Buss, 2015; Neel et al., 2016).

As individuals mature from infancy, a range of life tasks and biosocial goals unfold. Obvious ones are avoiding physical injury, predation, toxins, and diseases, while at the same time creating opportunities for resource gathering and reproduction (Parker, 1984). These goals may bring individuals into potentially costly and injurious competitive conflict with conspecifics who are going after the same resources. Therefore, solutions to the problem of conspecific competition have evolved over many hundreds of millions of years (Buss, 2015).

Most definitions of the word competitive define it as behavior designed to give individuals an advantage in the pursuit of resources. In his book, *The Selfish Gene*, first published in 1978, Dawkins outlined how evolutionary thinking shifted the analysis of competitive behavior from the individual organism to the gene. Important was the idea that specific strategies compete within individuals for expression and within populations for replication, for example, to be exploitative or helpful. In other words, genes build into the organisms that carry them, strategies, algorithms, and motivating systems that entice organisms to behave in ways that facilitate their reproduction in subsequent generations. Strategies may be more or less successful and therefore may have more or less chance of replication. However, in all populations, variations of strategic engagement are an outcome of evolutionary competition, thus giving rise to genetic variation, phenotypic variation, personality variation, and so on.

One of the big debating points in evolutionary science has always been how strategies for self-sacrifice and altruism could outcompete strategies for self-centered selfishness (Dawkins, 1978; Batson, 2017). Solutions to this puzzle emerged when the focus shifted to gene survival (inclusive fitness), rather than individual survival and hence, the focus of selection was kin and reciprocal altruism (Dawkins, 1978; Buss, 2015). Changing the focus of competitiveness to the gene, rather than the individual, opened up new ways of thinking about competitive behavior and the potential for altruistic, prosocial, and ultimately compassionate behavior to be rooted in competitive, survival, and reproductive strategies (Dawkins, 1978; Hardy and Van Vugt, 2006; Nesse, 2007; Fletcher and Doebeli, 2009; Gilbert, 2009, 2015).

What is passed from generation to generation is information for building biological systems that create strategies, motives, and algorithms to behave in ways that promote the survival and reproduction of the information that genes carry. Whether genes evolve to entice their carriers to behave aggressively or altruistically is dependent on reproduction frequency in populations and local contexts (Davies et al., 2012). Thus, for example, in mammals’, genes build motives and strategies for living in close proximity (groups and families), competing for resources, operating within social hierarchies, forming cooperative alliances, mating, and investing in offspring (Nesse, 2007; Davies et al., 2012; Buss, 2017). The proficiency by which animals enact these specific (social) strategies in specific contexts will result in reproductive fitness and therefore genetic success (Dawkins, 1978; Nesse, 2007). Hence, the distal and phylogenetic origins of many forms of behavior are the outcomes of strategic and phenotypic competition. In general then, although we think of competition operating between individuals, the drivers of competition are survival and reproductive *strategies* that are motivating and orientating brains to behave in certain ways and in certain contexts. Indeed, the human brain is full of competing strategies and motives, many of which are unconscious (Huang and Bargh, 2014) and many of which have genetic variations associated with them.

To return to our core theme, this article takes a broad-brush approach to variations in strategies underpinning social competition and highlights two different classes of survival and reproductive strategies, which can be labeled *prosocial and antisocial.* In their edited major overview of economic games, Brañas-Garza et al. (2016) utilize these dimensions suggesting that “Under the labels of ‘prosocial’ and ‘antisocial’ behavior we consider all those actions that help or hurt others, respectively” (p. 1). There is, of course, a long history to the study of antisocial behavior within criminal and psychopathological contexts. Subgroups of antisocial disorders have been identified such as the degree of callousness, aggressiveness, deceitfulness, lack of remorse, and even enjoyment from making others suffer (e.g., Piotrowska et al., 2015). Our use of the term is not to imply a specific personality disorder as such, but a dimensional element to social relating. For example, antisocial tendencies have been articulated for three personality dimensions of: Machiavellianism, narcissism, and psychopathy, known as the dark triad (Furnham et al., 2013). Here we are using the concept of antisocial strategies dimensionally to describe individuals who are primarily self-focused, manipulative, and threat focused, seeking to create inhibitory and submissive compliant states in those to whom they are directed (Caryl, 1988; Gilbert, 2000a, 2018; Sapolsky, 2017).

Prosocial strategies for “competitive resource acquisition,” seek to build coalitions and alliances and create secure low-level stress environments with a preparedness to care, support, and invest in others. Survival and reproductive success emerge through building cooperative alliances (Dunbar, 2016). In addition, there are major advantages to leaders creating relatively safe environments, which will impact on a range of physiological systems including stress and immune systems (Seppälä et al., 2017). The major constituents of prosocial motivation and behavior include concern for others’ wellbeing, empathic, cooperative and moral focused behaviors, joy at relieving suffering, distress at causing suffering, and capacities for remorse and guilt (Penner et al., 2005; Loewenstein and Small, 2007; Nesse, 2007; Brown and Brown, 2015; Ricard, 2015; Böckler et al., 2016; Richerson et al., 2016; Ewest, 2017; Seppälä et al., 2017). Importantly, one of the problems of the antisocial leader is that they can be seen as desirable and helpful leaders. Hence, individuals are attracted to them for all kinds of reasons to do with a sense of protectiveness or being made to feel special (Lindholm, 1993). Lipman-Blumen (2005) offers an in-depth analysis of why we are attracted to ‘toxic leaders’, to the extent that we are, and the serious problems arising from their attraction.

Chance (1988) outlined the social textures of primate and human groups that have aggressive versus friendly leaders. He described the former groups as “agonic” meaning that there is high stress within the group, fear of down rank threat, with potential conflict always just under the surface. By contrast, social groups can also be mutually supportive and facilitative and promote a different type of leadership, particularly in contexts of social safeness. He called these groups “hedonic,” where the relationships are friendly, sharing, and supportive. Hence, there are identified differences in primate and human groups in terms of their biopsychosocial manifestations. Sapolsky and Share (2004) describe an observation when a rubbish dump, where baboons tended to feed, became poisoned. Aggressive male baboons who tended to dominate the dump ate from the dump first and died off. This left the group with more females and largely unaggressive males. The basic structure of the group remained far more peaceful and affiliative for years to come.

While leader-follower motivational systems and behavior have evolved from earlier mammalian dispositions to form rank hierarchies of deference, partly to regulate potentially injurious competitive behavior (Barkow, 1980; Bernstein, 1980; King et al., 2009; Davies et al., 2012), more recent evolutionary adaptations have created hierarchies of attractiveness, such that audiences have opportunities to choose from, relate to, and learn from the more talented and able (Barkow, 1989; Gilbert, 1989/2016, 2007; Hardy and Van Vugt, 2006). Indeed, humans make judgments based on social comparison and ‘desirability’ ranking all the time, be it along dimensions of physical attractiveness, trustfulness, athletic ability, intelligence, and so on (Suls and Wheeler, 2013). Leaders depend on this “selection *via* attraction” in different ways. For example, within an organization, mid ranking leaders may need to appeal to those above them to be given a leadership role within an organization. Antisocial leaders can use what is called the *slime effect* or “upward licking, downward kicking strategies” (Vonk, 1998). In other contexts, leaders may try to stimulate attention and interest in themselves through oratory and displays of talents and abilities that followers may be inspired to follow (Lindholm, 1993). Leadership style is responsive to social context, as for example when organizations choose so-called tough leaders to make people redundant, or when organizations specifically seek out leaders who are likely to be moral and prosocial to those they lead. However, leaders also create social contexts (e.g., ones of threat and division versus ones of cooperation and mutual support, Van Vugt et al., 2008). For example, shifts to right wing politics may rise in contexts of threat, particularly job security and tribal threat which some leaders often stimulate (Duckitt, 2001; Janoff-Bulman, 2009). Indeed, it is well known that some human leaders rely on creating threat of external agents within a community, including influencing the social media (Sachs, 2012) in order to present themselves as strong leaders, protectors and saviors (Lindholm, 1993; Lipman-Blumen, 2005).

## Psychological Processes Facilitating Strategies

**Figure 2:**
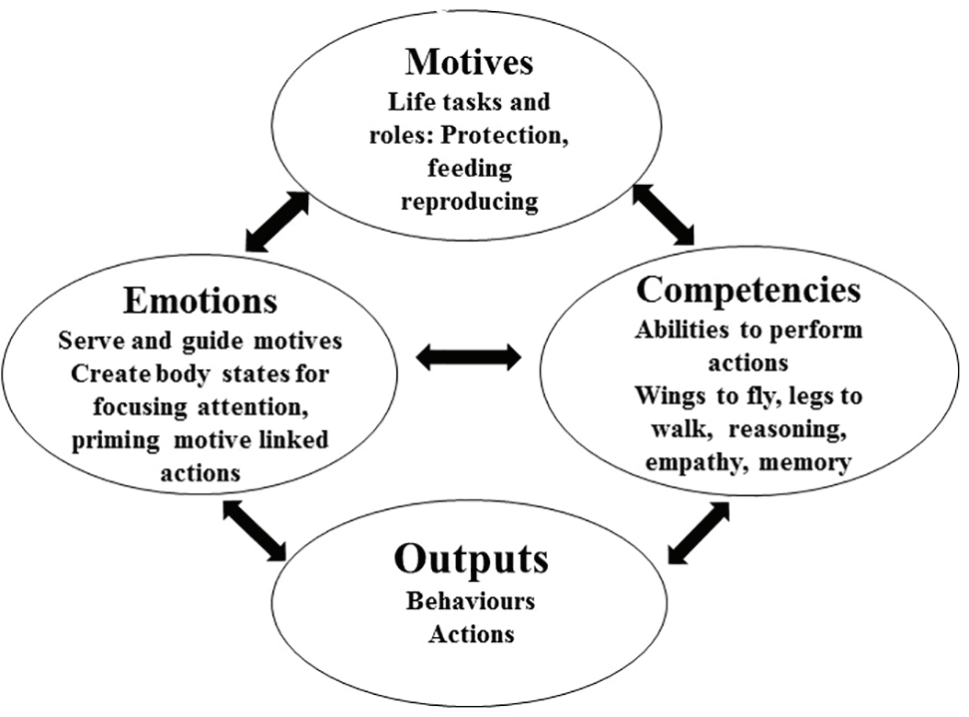
Interactions between motives, emotions, competencies, and output.

The move from gene-built strategies to the higher-level aspects of human psychology requires delineation of the basic systems that organize behavior. What systems do genes actually build in bodies and brains to facilitate the actions that facilitate their (genetic information) survival? There are four basic domains of functioning that facilitate strategic engagement. These are motives, emotions, competencies, and their outputs/behaviors and are depicted in Figure 2. We briefly consider each in turn.

**Motives** are stimulus sensitive and stimulus-seeking systems that guide actions and direct animals to survival and reproductive biosocial goals. Evolved motives include harm avoidance, finding and consuming food, competing for resources, status seeking, gaining and maintaining sexual/reproductive opportunities, caring for offspring, and forming alliances (Gilbert, 1989/2016, 1992, 2018; Deckers, 2014; Buss, 2015; Neel et al., 2016). Motives guide organisms over the long term, often over a lifetime. Individuals can vary in terms of how strongly they seek them out and respond to their success or failure. In addition, individuals can experience both conscious and unconscious conflicts between motives (Huang and Bargh, 2014). Humans create networks of submotives, as for example in order to gain status and resource access, students will study for years to follow a career, give up partying and take a job during vacations to get them through university. Human resource competition therefore, can have many submotives, but ultimately, they are rooted in evolved motives underpinning life tasks.

**Emotions** are notoriously difficult to define (Scherer, 2005). However, some researchers suggest that they are often short-lived physiological states that facilitate specific actions in specific contexts and are in the service of motives. Different researchers have classified the types and functions of different emotions in different ways (Ekman, 1999; Panksepp, 2004). However, Barrett (2017) suggests that emotions are far more variable and contextually sensitive. For our purposes, it is possible to identify three basic functions for emotion. The first is the function to detect and respond quickly to threats. This includes emotions such as anger, anxiety and disgust. These emotions can also be generated when there is an interruption or thwarting to resource-seeking goals. The second involves emotions that are energizing and rewarding and guide resource seeking. The third involves emotions that are associated with rest and digest and are calming, soothing, and contentment based (see Gilbert, 2009, 2014 for details).

The link between emotions and motives is complex. Emotions and feeling states can also become motives themselves. For example, we can be motivated to create pleasure and excitement for its own sake, hence the problem of addiction. Competitive behavior will be partly regulated by the emotions that are generated along the way. Invigorated or attenuated competitive behavior may relate to the emotional experiences associated with success or failure. There can be individual variation in regard to the textures and intensity of emotional responses to success or failure. In psychotherapy and leadership training, emotion regulation (training) is often central. Although threat-based emotions are often the focus for emotion regulation training, attention has also been directed to the importance of activating and regulating positive emotions, both those that are activating, as in joy and excitement, but also those that are calming, soothing, and affiliative (Gilbert, 2009, 2014, 2017b).

**Competencies** are capacities to perform functions. For example, birds use wings to fly, mammals use limbs to move around on, and humans have competencies for sophisticated hand and finger use, hence the ability to play Rachmaninoff piano concertos. However, it is not just competencies of dexterity, but extraordinary integrated abilities of memory, cognitive and self-aware competencies, with a self-identity that wants to play, along with dedication to practice over many years that enable us to perform such feats. Driving a car too involves extraordinary feats of cognitive integration, being able to alter behavior moment by moment, over many hours, as we change gears, go fast and slow according to traffic flow, while having a conversation with the person next to us or thinking about what we are going to say at the meeting that we are driving to. Other obvious, new competencies that separate humans from other animals are the extraordinary evolution of cognitive competencies over the last two million years. These have changed the nature of motivation and emotion regulation (Gilbert, 2009; Dunbar, 2016; Suddendorf, 2018). Evolved psychological competencies include capacities for empathy, language and symbol use, reasoning, imagining, planning, metacognition, anticipating, memory, creative and systemic thinking, but especially capacities for integrative multi-dimensional action as in driving on the motorway. These far exceed any other species. In addition, humans have levels of objective self-awareness that also far exceeds anything in any other species as far as we know. This means that humans can engage in behavior intentionally. For example, we can choose to exercise and get fit intentionally or we can choose to practice the piano with the intention of being a good player. No other animal can intentionally change themselves. We have insight into the consequences of our behavior that animals do not have, meaning that even if we are motivated to behave in a certain way, our perception of the consequences may significantly facilitate or inhibit that behavior. We can choose to gain an insight into our minds, train our minds, and develop skills and emotion regulation.

Another new evolved competency is a form of conscious awareness, “a consciousness of being conscious” that underpins knowing intentionality. This is our ability to be an observer of our minds and underpins the ancient focus of mindfulness (Gilbert and Choden, 2013). Indeed, part of civilization, as Freud argued, is learning to become aware of and then appropriately inhibit our basic harmful impulses for greed, sex and aggression. These are phenomenally important competencies that can regulate how competitive strategies are played out. They can be fundamental to leadership style too.

Our new brain competencies have completely changed the dynamics and consequences of motivational systems. For example, the invention of contraception has changed fundamentally the link between sexual behavior and reproduction. Our capacities for international travel provide huge opportunities for gene mixing, which has never been seen before. Medicine has prevented vast numbers of people dying that would not then have contributed to the gene pool. The flow of knowledge *via* modern communications systems has changed our environment forever. However, our new brain competencies are both a blessing and a curse.

They are a curse when we recognize the terrible dark side to these competencies. We can use our intelligence in pursuit of ruthless self-interest and tribal conquest. Basically, our new brain competencies are hijacked and recruited into the fulfillment of basic motives and algorithms that are many millions of years old. The degree to which we are “scripted” by our phenotypes was a theme well explored in the TV series Westworld. Competitive behavior takes on whole new textures with an ‘intelligent’ human mind. Tragically, humans are probably one of the most sadistic and nasty species to have ever walked the earth. We have engaged in the most horrendous wars, invented the most horrifying tortures, industrially killed millions (the Holocaust), have enslaved billions, and even take entertainment from watching cruelty and slaughter (the Roman games). Human history is soaked in the blood of many billions of people who have suffered horrifying fates at the hands of other humans. This is indeed a tragedy because, although certain survival and reproductive strategies, such as tribalism and dominant aggression, operate in many other species, they lack our various competencies of intelligence that makes us potentially very vicious and dangerous. Yet to highlight the fact of just how multiple and complex motivational systems are, it is clear that we equally have the potential to work with other strategies for caring and investing in others and generate professions such as medicine and teaching (Gilbert, 1989/2016, 2009, 2018). Although religions are often hijacked by dominant leader males, to play out old tribal and sexual strategies, they also have within them the desire to combat our dark side by finding ways to treat others as equals and work for peace and compassion (Plante, 2015; Ricard, 2015). The problem is that caring for others can be costly, and therefore, in everyday life, there may be natural boundaries around it (e.g., focused on kin, friends, and allies) that we have to overcome using our intelligence (Loewenstein and Small, 2007).

**Behaviors** are primarily the outputs and manifest expressions of these processes. They can be worked on directly. For example, individuals who practice doing things they may be fearful of can lose their fear and come to enjoy them. Practicing behaviors to enable us to become good at something may increase our pleasure and sense of mastery. With that comes increased motivation. Human education is based on this fundamental capacity of “guided learning” with behavioral practice.

## Physiological Systems

The last 10 years have seen increasing research into the physiological basis for different motivational systems and emotions. For example, hostile competitive behavior works through very different peripheral and central physiological systems compared to prosocial behavior (Seppälä et al., 2017). Prosocial behavior has a range of important physiological effects, both in the expresser and in the receiver (Colonnello et al., 2017; Mascaro and Raison, 2017; Seppälä et al., 2017). Research suggests that there are several different brain areas involved in prosocial compared to antisocial motivation and behavior, including reward-related areas of the brain such as the ventral striatum (Harbaugh et al., 2007; Tabibnia and Lieberman, 2007; Vrtička et al., 2017).

In general, different forms of competitive behavior and leadership styles reflect unique patterning of motives, emotions, competencies, and behaviors, and these can be traced back to genetic and epigenetic algorithms through to (neuro) physiological infrastructures. It is this patterning that forms the human phenotypes for social relating, including styles of leading and leadership.

## Social Mentalities

The patterning of motives, emotions, and competencies in social interactions creates social mentalities. A social mentality refers to the complex interpersonal dances in reciprocal interactions that result in role formation around specific biosocial goals (Gilbert, 1989/2016, 2000b). As individuals interact, moment by moment, they may be processing fast changing stimulus presentations *via* different channels of communication (e.g., verbal and non-verbal). Their ability to do that, in order to co-create a specific role relationship, has been referred to as a social mentality (Gilbert, 2014). Hence, leaders can be assessed in terms of their motives, emotion engagement and coping, competencies such as for mindfulness and empathy, and their actual behaviors. Leadership training should address *all* these domains. All forms of competitive behavior require actors to be able to engage in certain interpersonal dances in order to gain and maintain their positions and successful role enactments. These reciprocal processes can operate at the physiological, non-conscious level.

Competitive motives (social mentalities), like all motives, require two basic processes (Deckers, 2014; Gilbert, 2014; Buss, 2015)*. Stimulus detection, seeking,* and *stimulus-meaning* are part of the first process. The second is appropriate *responding*. Each requires a number of competencies that are species specific and motive specific. For example, conspecifics will display different social signals to each other and will respond to those signals quite differently, according to whether the role formation is going to be for biosocial goals of courting and breeding, competing for resources (threatening and submitting), building alliances (as in grooming), or forming caring attachments to offspring. Hence, conspecifics engage in various “dances of social interaction” to enable specific, role focused relationships to form, enabling the securing of biosocial goals. A rough description might be: *A social mentality creates interpersonal dances for the formation of role relationships to pursue biosocial goals (e.g. status, mating, offspring care), recruiting socially intelligent competencies in the service of the social motive* (Gilbert, 2017a,b, p. 41).

The idea of an ‘interpersonal dance’ is useful because it represents a dynamic, reciprocal, and interactional flow that co-regulates emotions, motives, behaviors and physiological states in participants. Social signals seek to communicate information about some aspect of the self, such as an intention or emotional state, and thereby seek to form particular social roles that have specific functions. For example, a courting display, a dominant display, a submissive display, and a friendly display are all invitations to form a certain type of role relationship. These will also have important impacts on the physiological patterns activated in participants.

The study of competitive behavior, as it operates through leadership, is therefore partly a study of display behavior that functions to create various states of mind in those who are being displayed to (Barkow, 1989; Lindholm, 1993; Gilbert, 2018). First and foremost, dominant displays are displays to capture attention. Indeed, the primatologist Michael Chance pointed out that dominant hierarchies are also attention hierarchies related to both the quality and type of attention conspecifics pay to each other (Chance and Larsen, 1976; Chance, 1988). It is through this mechanism of attention regulation that an individual may seek to create, in the mind of another, desires for closeness or distance, a sense of safeness or fear and threat, defect, submit, or follow, and desires for cooperation or conflict. Obviously, leaders who are not able to regulate the attention and physiologies of those they lead are going to be less effective than those who do. Not surprisingly then, some politicians study the physiological impacts of some of their messages and speeches on selected audiences. The question is, what is being stimulated in potential followers or subordinates that make them pay attention to leaders and orientate the behavior in support of what the leader offers? In addition, is following compliant or submissive behavior based on voluntary, approving or involuntary, fear-based motives?

## Intra and Intersexual Competition

To move deeper into the evolved mechanisms underpinning competitive behavior, along the dimensions of antisocial (threatening hostile) and prosocial (friendly supportive), we now take a more detailed look into different types of competitive behavior linked to reproductive strategies. At the level of individual organisms, there are two basic types of competition (Davies et al., 2012). One is scramble competition, where individuals simply try to take as much of a resource as they can, but their behavior does not have an influence on others going after the resource. For example, birds feed on a field of wheat. However, if the resources are scarce (there is only one piece of bread on the lawn), then direct contest competition occurs whereby to have access and control of resources require individuals to challenge each other, creating winners and losers (Parker, 1984; Davies et al., 2012). In some forms of competition, this is called a zero-sum game, meaning that the benefit to one is matched by the loss of another. Not only is losing access to the resource important, but there are also potential injury costs from the conflict itself. Hence, as noted below, for species who live in groups, there could be a constant challenging for resources when scarce (e.g., sexual opportunity), which would be costly. Different hierarchies partly regulate this (Bernstein, 1980; Gilbert, 2000a; Fournier et al., 2002).

Reproduction and replication of genetic information, generation to generation, are obviously central to the whole evolutionary process. Hence, one of the main drivers of evolution for a range of social motives has been set by sexual competition. There are two quite distinct and different forms of it: intra and intersexual competition, which require different strategies underpinned by different attention sensitivities, motivations, behaviors, and physiologies (Buss, 2015, 2017). The interplay of these strategies and social mentalities textures a lot of human social life.

**Intrasexual competition** is based on competition between same gender members. It evolved primarily with the ability to deter, subdue, and/or inhibit competitors. Not all such competition is aggressive, as, for example, in some species dominant females secrete hormones that suppress ovulation in competitors. Generally though, sexual competitive behavior involves social mentalities of complex interactions where the outcome of the interaction determines the winner and the loser (or those that do better than others). When it is aggressive, behaviors are referred to as *ritualistic agonistic behavior* (RAB). It is ritualized to the extent that although injuries can be inflicted, the form of fighting is very different to predation and killing. Indeed, different species have different threat and submissive signals that are involved in the ritualistic agonistic “dances” (Gilbert, 2000a). These evaluative systems are rooted in what is called *resource holding potential* (Parker, 1974) and expressed *via* RAB. These ritualistic displays facilitate social comparison and enable competitors to weigh each other up as to the likely outcome of a conflict, forming dominance subordinate hierarchies where subordinates are prepared to recognize their subordinate status and not engage in conflict, but to submit and escape quickly to prevent injury (Bernstein, 1980; Caryl, 1988; Gilbert, 1994, 2000a). In addition to basic fight and flight defensive behaviors, submissive displays have evolved to turn off aggressive attacks in the dominant. For example, primates typically crouch and avert eye gaze, and wolves roll onto their back and bare their throat, which typically ends hostilities (Gilbert, 2000a; Davies et al., 2012). Hence, those who evaluate that they have the ability to win will escalate, while those who feel that they are likely to lose or be injured will deescalate. Sometimes this is called the Hawk and Dove strategy (Caryl, 1988). Clearly, some forms of human competitive behavior and leadership engage in RAB, where voice tones, non-verbal communication, and outright threats, even violence, are used to force compliant submission in subordinates and injure and kill competitors; the psychology of tyrants. Fournier et al. (2002) have also shown that in human contexts, when subordinates are criticized by more dominant individuals, they tend to be submissive, whereas when subordinates criticize those above them, the more dominant individual becomes quarrelsome and counter attacks. Indeed, antisocial leaders do not respond well to criticism. While females are less physically aggressive, they can also use threat and intimidation as tactics of competition suppression to potential challengers (Davies et al., 2012).

Importantly however, when competitive behavior and obtaining dominant positions require alliances, then the challenge is to elicit support from allies (Barkow, 1989; Boehm, 1999). In these contexts, the would be dominant *needs to be attractive* in some way to potential allies and supporters. Hence, intersexual competition (ways of gaining access to resources and reproductive opportunities) needs to be quite different. Potential allies need to see some benefit and feel sufficiently safe with each other, rather than just be held in fearful compliant mental states.

**Intersexual competition** is related to competing to attract mates and be accepted as a breeding partner. Although in various species, forms of mating can be coercive by males (and in humans this is represented as rape), intersexual competition is also dependent on attraction (Barkow, 1989; Etcoff, 1999; Hardy and Van Vugt, 2006; Buss, 2015). Many traits including for example, bird colors and pheromones are evolved attractors (Lyon and Montgomerie, 2012). Humans created the fashion industry and spend much time and money on their appearance or status displays. This can be not only an enticement to sexual partners but also a competitive signal to people of the same gender. In competing *via* attractiveness, there is a limit to aggressiveness and threat as a successful reproductive strategy, particularly where females or allies are able to reject aggressive individuals and subordinates can gang up and dispose aggressive-dominant individuals. This was likely in certain early, small hunter gatherer human groups (Boehm, 1999).

Eliciting support and friendly social signals from others and engaging in prosocial behavior have also evolved to have enormous physiological benefits, as noted above (see Seppälä et al., 2017; Petrocchi and Cheli, 2019 for reviews). Indeed, the evolution of caring, sharing and cooperative behavior, including language, would have only been possible if individuals felt safe enough with each other, to get and stay close to engage in sharing behavior, indicating the importance of physiological systems that are responsive to cues of care, friendship, and social safeness (Gilbert, 1989/2016, 2015). The experience of feeling socially safe in one’s social environment is a significant predictor of a range of health-linked outcomes (Kelly et al., 2012). For females, kin-based supportive networks and alliances are extremely important for support of offspring and well-being. Female matriarchy and leader females can have a significant impact on the group as a whole. This is particularly true for human females (Taylor, 2006; Hrdy, 2011). Social affiliation, therefore, becomes an important evolved trait that can offer competitive advantage, for both survival and reproductive (Dunbar, 2010, 2016).

Unfortunately, this does not mean that aggression is not used in intersexual competition across the genders. Indeed, conflict between female and male reproductive strategies have been well discussed in the evolutionary literature (Buss, 2015, 2017) and noted some time ago by Wilson and Daly (1992) in a provocative, insightful chapter, *The Man Who Mistook His Wife for a Chattel.* Tragically, men can be extremely aggressive to women in an effort to mate without responsibility for subsequent investment (e.g., rape). Threats and forms of jealousy are also utilized to ensure sexual loyalty, amongst other things. In many species, males try to limit and control female mating opportunities, often aggressively. There are many ways of intimidating partners to comply with the competitive reproductive strategies of the male. Religions have also played their role in creating cultural contexts portraying women as subservient to men (Plante, 2015). The epidemic of domestic sexual violence is evidence that aggressive and antisocial strategies are prevalent in human males. The size of the problem can be illustrated by a major World Health Organization study by Garcia-Moreno and her colleagues, which was conducted in 15 sites in 10 geographically and culturally diverse countries (Garcia-Moreno et al., 2006). To quote from their own findings’ summary:

Twenty-four thousand and ninety-seven women completed interviews, with around 1500 interviews per site. The reported lifetime prevalence of physical or sexual partner violence, or both, varied from 15 to 71%, with two sites having a prevalence of less than 25%, seven between 25 and 50%, and six between 50 and 75%. Between 4 and 54% of respondents reported physical or sexual partner violence, or both, in the past year. Men who were more controlling were more likely to be violent against their partners. In all but one setting women were at far greater risk of physical or sexual violence by a partner than from violence by other people. (p. 1260)

As the authors point out, violence against women is widespread, has often been ignored, is culturally variant, and desperately needs to be addressed *on an international scale.* It is well known that domestic violence is linked to alcohol abuse, male low self-esteem, and even one’s football team losing! In many species including humans, dominant males often exploit their power positions for sexual access to females and engage in harassment. The strategies and algorithms of mating they use are not always pleasant or moral. As noted below, this is why regulating social contexts and moral development is central, given the evolved dark side of the human mind (Gilbert, 2018). The problem with sexual violence is risk of injury to the female and thereby reducing reproductive success. Hence, one of the benefits of compassion and caring and inhibiting aggressiveness is avoiding injury to children and female partners. If the algorithms for compassion are aggression regulators, and they are not working, maybe because of early life histories and contextual cues, then people may revert back to these older strategies. Therefore, mindful compassion training throughout all sections of society is of vital importance. Indeed, there are wide cultural variation in the acceptance of aggression against women, children, and in defense of honor. In other words, although there are potential algorithms for males to behave in dominate aggressive ways, there are also ways in which these can be inhibited from expression. Evolved algorithms are not the equivalent of the fates. One function of leadership today may well be creating, promoting, and teaching how to create contexts for affiliative, rather than hostile and exploitive sexual relating.

For non-human primates, threat will create social spacing between conspecifics. Indeed, when primates are free moving, although aggression can take place, primate females are less subjected to the kind of systematic harassment, exploitation, and violence that human females can be. For the most part, they gather their own resources, are not dependent on the males for resources, and can escape from threats. It is partly because we have created cultures where men can trap women in relationships. They live in isolated boxes called houses and are vulnerable to loneliness and disengagement from supportive networks. Indeed, loneliness and disconnection from supportive networks are the fastest increasing mental health risks. In addition, women are often dependent on men for resources (supported by religious dictates) and thus exist in contexts from which they cannot escape. No other female in nature is trapped and constrained like this, disconnected from important supportive female networks and reliant on males for survival. This is one of the (many) tragic downsides of human culture. Into this grossly abnormal environment, we grow our children (Narvaez, 2017). Increasingly, we need political leaders who understand our minds as evolved, with inbuilt needs, motives, and algorithms that cultures can operate on for better or worse. Self-focus competitiveness does not sustain us. The evidence is overwhelming that it is our prosocial relationships and a sense of living in supportive and caring communities that is crucial to wellbeing (Gilbert, 2009, 2018; Kelly et al., 2010). The problem however, is that antisocial leaders are more interested in promoting sensitivity to threat, increasing focus on individual competitiveness maintaining traditional sexual stereotypes and appealing to and stirring tribal, intergroup conflict.

## Threatening Versus Attracting

Throughout the article, we have been highlighting that engaging in social competition using different strategies can be labeled as antisocial or prosocial. The former is primarily tactics of intimidation and threat, whereas the latter is non-threatening and seeks to be esteemed and chosen for their positive/helpful attributes. These are obviously not mutually exclusive, and people can move between them, even within the same relationship and according to the context. Even if people love each other, under conflict and particularly when anger arises, there can be a shift toward more aggressive strategies for winning the conflict. These two strategies are depicted in Table 1.

**Table 1:** Strategies for gaining and maintaining rank-status in social roles.

Strategy	Aggression	Attractiveness
Tactics used	Coercive Threatening Authoritarian	Showing talent Show competence Affiliative
Outcome desired	To be obeyed To be reckoned with To be submitted to	To be valued To be chosen To be freely given to
Purpose of strategy	To inhibit others To stimulate fear	To inspire, attract others To stimulate positive affect

*From Gilbert and McGuire (1998)*.

As noted then, under conflict, we can revert to ritualistic antagonistic behavior (RAB) and resource holding potential (RHP), displays of fighting, and potentials for winning conflicts through threat and aggressive means. In humans, these social signals may be ones of raising one’s voice, facial displays of anger or contempt, and verbal content that is shaming. Primatologist Chance (Chance and Larsen, 1976; Chance, 1988) noted that dominance hierarchies are also attention hierarchies. Using his approach (Chance, 1988, personal communications), Gilbert (1989/2016, 1992, 1997, 2007) suggested that social hierarchies could also be considered as arising from social attention holding power or potential (SAHP), which could be contrasted to RHP (Gilbert et al., 1995). SAHP was basically linked to various forms and displays of talent that could have competitive advantage. What conspecifics give their social attention to depends on the nature of the group. In cooperative human groups, there may be various forms of skill and attributes that people wish to copy, admire, or partake of. When talents are scarce, groups may offer resources to secure them, for example, noted surgeons or desired actors and actresses. One of the important domains for SAHP is social reputation, whereby positive reputation facilitates cooperative and conspecific helpful behaviors, whereas damaged, shamed, or poor reputations are associated with social exclusion and rejection (Gilbert, 1997; Sznycer et al., 2016). Barclay (2004) suggests that individuals may act more altruistically, even though they may not benefit directly (referred to as indirect reciprocity) to increase their reputation and perceived trustworthiness. By behaving altruistically in certain contexts, they may be perceived as more attractive and able to secure increased status and prestige within groups (Hardy and Van Vugt, 2006). As groups benefit from their presence, they will continually reward them.

In general then, human competition has become increasingly focused on the needs to be chosen by audiences who are selecting on the basis of competency in and for specific roles, such as friend, ally, sexual partner, or employee (Barkow, 1989; Sapolsky, 2017). Be it in the school sports team, joining a supportive peer group, being wanted as a sexual partner, being chosen for employment, much of human social competition involves impression management and avoiding being marginalized or rejected. Indeed, perceived difficulties in being able to compete for social place and status, and feeling inferior and marginalized, are related to problems such as depression, loneliness, and anxiety, particularly in the young (Gilbert et al., 2009; Crocker and Canevello, 2012; McEwan et al., 2012).

In fact, many writers have seen much of human competition centered around the need for recognition, status and approval, because it is a gateway to many forms of beneficial relationship (Cheng et al., 2014). Fukuyama (1992) gave this concise historical overview when he writes:

The concept underlying “recognition” was not invented by Hegel. It is as old as Western political philosophy itself and refers to a thoroughly familiar part of the human personality. Over the millennia, there has been no consistent word used to refer to the psychological phenomenon of the “desires for recognition”: Plato spoke of thymos, or “spiritedness,” Machiavelli of man’s desire for glory, Hobbes, of his pride or vainglory, Rousseau, of his amour proper (self-love), Alexander Hamilton of the love of fame, and James Madison of ambition, Hegel of recognition, and Nietzsche, of man as the “beast with red cheeks”. All of these terms refer to that part of man which feels the need to place value on things – himself in the first instance, but on the people, actions, or things around him as well. It is the part of the personality which is the fundamental source of the emotions of pride, anger, and shame, and is not reducible to desire, on the one hand, or reason on the other. The desire for recognition, is the most political part of the human personality because it is what drives men to want to assert themselves over other men and therefore into Kant’s condition of “asocial sociability”. (p. 162–163)

Etcoff (1999) refers to particular domains of this type of competition as *Survival of the Prettiest.* She reviewed considerable evidence that physically attractive people and those with attractive personalities or likeability tend to do better in many aspects of life such as better career prospects, better earning, and lesser sentences for minor crimes. In addition, just as animals can calculate their own RHP, humans can calculate their own SAHP that can underpin feelings of self-esteem and self-worth or its contrast, shame (Gilbert, 1997, 2007). The concept of SAHP also sought to capture abilities to create positive images in the minds of others and be a positive attractor. This switch in the dynamics of social competition, from threat based to approval-based competition (Barkow, 1980, 1989), also changed the qualities for social comparison (Gilbert et al., 1995) and status seeking (Cheng et al., 2014) and made shame a central social evaluative concern for humans (Gilbert, 2007; Sznycer et al., 2016). Indeed, hostile conflict no longer needs to be physical, it could be attacks on social standing by shaming and undermining an individual’s SAHP and reputation and thus cutting them off from potential helpful cooperative alliances and liaisons. Antisocial leaders use these tactics all the time to “dig the dirt” on competitors. One of the concerns in politics today is now not about promoting positive qualities of one’s policies, but constantly finding ways to undermine and shame opponents (Sachs, 2012). Thus, the politics and leadership contests are ones of derogation rather than promotion.

Other ways of considering these two basic dimensions of antisocial and prosocial leadership have been suggested but labeled differently as *dominance versus prestige* (e.g., Cheng et al., 2013; Henrich et al., 2015; Maner, 2017). These models view dominance in terms of the more aggressive styles of leadership linked to old mammalian social rank formation strategies that use variations in what is noted above as and been termed in ethology as *ritualistic antagonistic behavior* (Parker, 1974; Caryl, 1988; Gilbert, 1994, 2000a). They are particularly linked to the dark triad personality styles namely Machiavellianism, narcissism, and psychopathy (Furnham et al., 2013; Maner, 2017). This use of the term dominance combines the *motivation* for dominance seeking with particular emotional dispositions and *personality traits* of aggressiveness, manipulativeness, and callousness. However, it is possible to be high on dominance seeking without those particular personality traits. For example, dominance seeking has been linked to bipolar disorder and forms of hypomanic personality disorder, but individuals with these disorders are not necessarily callous or manipulative (Johnson et al., 2012). Indeed, individuals may strive for “dominant positions” for all kinds of reasons, not necessarily using threatening tactics or behaving callously. Cislak et al. (2018) highlight that power seeking when it is to control others tends to be associated with aggressive and manipulative strategies, whereas power seeking when it is associated with personal freedom and to be free of the control of others is negatively associated with those strategies.

Other authors have pointed out that dominance is really an outcome of a competitive interaction. For example, Bernstein (1980) notes that you cannot tell if an animal or human is dominant by just looking at their behavior. It is the *behavior of the subordinates around them* that give indications of the types of relationships arising. Individuals may want, strive for, and behave in dominant ways; however, if conspecifics simply ignore them or even attack them, then obviously dominance does not arise (Gilbert, 2000a). Therefore, that competitive strategy fails. In addition, it is the behavior of subordinates that send signals to the dominant, which impact on various physiological systems. When dominant monkeys are put behind a one-way mirror and they can see subordinates, but subordinates cannot see them, hence subordinates stop sending submissive signals, there are major changes in the physiology of the dominant (Gilbert and McGuire, 1998).

In contrast to dominance seeking, Maner (2017) argues that prestige styles are primarily human, focusing on needs for approval, displays that attract and stimulate approach behavior rather than threatening others, and agreeableness as a personality style, with a fear of negative evaluation indicating underlying social anxiety. Again, however, many animals display themselves in non-aggressive ways to attract sexual or other interest. Therefore, prestige can have many different meanings. It is related to seeking a certain kind of approving (rather than fearful) attention from audiences and stimulate approach rather than avoidance behavior. Despite these subtle differences, there is growing agreement that some forms of status seeking and leadership are prosocial and some are not and can be quite harmful (Gilbert 1989/2016; Sachs, 2012; Cheng et al., 2014).

As noted the focus on competing for recognition and attractiveness was also articulated as a status prestige-seeking alternative to aggressive competitive behavior by the anthropologist Barkow (1975, 1980, 1989) and links to SAHP (Gilbert, 1989/2016; Gilbert et al., 1995). The issues here are whether one should combine a *motive* for say approval seeking or status seeking (Anderson et al., 2015) with *personality traits* such as agreeableness, need for affiliation, and fear of negative evaluation as an outcome. Maner (2017) suggests that the personality trait of agreeableness is part of prestige-seeking leadership styles. The problem is that agreeableness is a tricky concept, because context plays a big role (Judge et al., 2012). Indeed, agreeableness has a downside when linked to being submissive to avoid conflict. Bègue et al. (2015) found that agreeableness was associated with compliance in Milgram type experiments where participants were asked to behave powerfully to another. Prosocial leadership, however, requires courage to stand against immoral or harmful actions, and courage is essential to compassion (Gilbert, 2009; Ewest, 2017). Hence, prosocial leaders do not always behave agreeably to all audiences.

Although we can identify many variations in the way humans compete for social attention, status and leadership roles, it is not always clear exactly what they are competing for (Cheng et al., 2014). While some forms of competitive striving are linked to desires for dominance, control over others, and a sense of superiority (Martin et al., 2014) and to some degree greed (Van Kleef et al., 2008), many forms of competitive behavior and leadership styles are related to the fears of inferiority and the avoidance of being marginalized, subordinated, and rejected, basically to social threat. Indeed, there is a difference in competitive behavior designed to exert control over others versus avoiding others exerting control over the self or being marginalized.

Gilbert et al. (2007) developed measures that distinguish between insecure and secure competitive striving. Insecure striving and competitive behavior are linked to fears of failure, active rejection, being passed over or marginalized, losing out, missing advancement opportunities, and depression and anxiety. By contrast, secure competitiveness was not linked to the fear of failure nor to worries of rejection in the face of failing. The study found that insecure competing was associated with hypercompetitive attitudes (*r* = 0.57) and insecure attachment (*r* = 0.56) (Gilbert et al., 2007). In a recent study Basran et al. (2019) found that striving to avoid inferiority was significantly associated with narcissism, ruthless self-ambition and hyper- competitiveness, indicating that these antisocial traits may well be rooted in fears of being marginalized and rejected.

## Prosocial and Antisocial Styles of Competition, Leadership, and Personality

There are many dimensions pertinent to the study of how people compete for resources and try and influence each other in their own self-interest, both within intimate relationships and in wider leadership contexts (Cheng et al., 2014; Buss, 2015, 2017). Interestingly, studies have shown personality differences in dominant baboons too and indeed in all primates that live in troops and groups. Sapolsky (1990) and Ray and Sapolsky (1992) described some males as insecure dominant. These individuals seem moody and unpredictable, often pick fights and are more likely to be aggressive with females. By contrast, secure dominant males respond aggressively to threats upon them but do not provoke them and were generally more affiliative with females and subordinates and engage in grooming with other group members. A range of physiological profiles distinguished them too. Human studies of personality types are far more varied, but they have been explored in regard to leadership, including the dimensions of the big five personality traits of agreeableness, openness, extroversion, neuroticism, and conscientiousness (Judge et al., 2009). Other descriptions of personality such as Machiavellianism, narcissism, and psychopathy, often referred to as the dark triad, also pertain to competitive strategies and leadership style (Furnham et al., 2013; Muris et al., 2017; for reviews see Judge et al., 2009).

Another dimension that integrates forms of competitive behavior and adds further insight into antisocial leadership styles is social dominance orientation theory (Ho et al., 2015). This approach focuses on dominance in terms of group dynamics and sociocultural beliefs. Ho et al. (2015) offer a major overview of the important findings from this research tradition, as well as distinguishing two dimensions of social dominance orientation:

The dominance dimension is characterized by support for overt oppression and aggressive intergroup behaviors designed to maintain the subordination of one or more groups, whereas the anti-egalitarianism dimension entails a preference for intergroup inequalities that are maintained by an interrelated network of subtle hierarchy-enhancing ideologies and social policies. (p. 1004)

Ho et al. (2015) point out that social dominance-orientated leaders, in both politics and religion, tend to be socially divisive, seek to privilege their own group, and accentuate the external threats to their group. Martin and Heineberg (2017) reviewed the relationship of these traits with leadership styles. The evidence is, as one would expect, that social dominance orientation is highly correlated with the more antisocial aspects of leadership, being less empathic, less warm, less compassionate, and more aggressive, whereas prosocial leadership is associated with the opposite.

Duckitt (2001) draws attention to the way some concepts of dominance relate to older concepts, such as the authoritarian personality first described by Adorno et al. (1950). These are individuals who believe in the regulation of behavior through power hierarchies, advocate aggression to non-compliant subordinates, and support social inequalities. They are also attracted to leaders who endorse these punitive leadership styles. There are differences between them, however, in which authoritarian personalities see the world as more threatening and dangerous than social dominance orientation people may do, and are more likely to take their legitimacy from appeals to religious “higher powers” who must be obeyed (“God has ordained that …”). In the eyes of the “higher powers,” they believe in their own “chosen-ness,” righteousness, and specialness. Commonly what “God” has ordained, turns out to favor male sexual competitive strategies and group claims on resources or territories.

Antisocial leadership styles are commonly linked to criminality, but increasingly, this is recognized to be a very limited focus (Millie, 2008). As noted in the introduction, antisocial motivation and behavior can be seen dimensionally pertaining to a general lack of caring interest for others, preparedness to cause harm for personal advantage and poor moral codes. However, these dispositions operate throughout populations (Furnham et al., 2013; Gilbert, 2018). Indeed, it is relatively easy to entice people to behave in harmful ways to others (Kelman and Hamilton, 1989; Zimbardo, 2006). In larger groups, however, antisocial leaders who are related to at a distance, rather than in direct interpersonal contact, can be seen as attractive, and as being strong protectors to threats (from crime, immigrants, dissidents, or other groups; Lipman-Blumen, 2005). History shows that many political regimes have been dominated by antisocial leaders who are not averse to using state-sponsored intimidation including torture and murder. Moreover, tragically even when their supporters may know their leaders are doing this, they still support them (Lindholm, 1993). What seems to be central to this dimension is their threat sensitivity and readiness to engage in antisocial behavior.

On the other hand, prosocial strategies for competitive behavior seek to build coalitions and alliances and create secure low-level stress environments with preparedness to care, support, and invest in others (Ewest, 2017). The major constituents of prosocial personalities include a range of motives, emotions, and competencies such as concern for others, empathic awareness of the impact of their behavior on others and a moral focus (Penner et al., 2005; Loewenstein and Small, 2007; Brown and Brown, 2015; Böckler et al., 2016; Richerson et al., 2016). Prosocial leaders overlap with what has been called servant leadership (Spears, 2010) were leaders primarily focus on the growth and development of those they lead. In addition, prosocial leaders focus on, and will try to work against, inequalities, promote social fairness, and regard aggressive means of control as undesirable (Ewest, 2017; Worline and Dutton, 2017). They are more likely to be authoritative rather than authoritarian, confident, but not hostile in demonstrating their skills and knowledge, while being appreciative of the skills and knowledge of others.

Importantly, what is called prosocial leadership overlaps with what we can call compassionate leadership (Gilbert, 2018). This is because compassion is rooted in mammalian caring motivational systems (Mayseless, 2016). Caring becomes compassionate when it is guided by more recent evolved cognitive competencies that give rise to knowing awareness, knowing intentionality and insightful empathy (Gilbert, 2017a,b, 2018). Compassion is a (knowing and intentional) sensitivity to suffering in self and others with a thoughtful, wise orientation to prevent and alleviate suffering (Gilbert and Choden, 2013). In addition, compassion carries the self-identity to not carelessly or purposely cause suffering. There is increasing evidence that compassion training can influence a range of physiological processes and orientations toward altruism (Boyatzis et al., 2006; Weng et al., 2013). Prosocial leaders can also morally contextualize their activities in the wider world. They have what might be called an expanded moral compass (Ricard, 2015; Crimston et al., 2018).

Prosocial and antisocial leadership styles (be they in parents, teachers, managers, or politicians) may differ quite significantly on the various competencies of compassion and prosocial behavior. For example, they may differ on the degree to which they are sensitive to and can tolerate their own, and thus other people’s distress and emotions in general (Shirtcliff et al., 2009), some may be alexithymic (Gilbert et al., 2013), while others struggle with empathic competencies (Baron-Cohen, 2011). We stress that these are competencies and should be clearly distinguished from motivation, because people can be highly motivated to be caring, but can struggle with knowing how to be caring. Others can be empathically competent, but are motivated by more selfish goals and, like those with psychopathic temperaments, have little interest or motivation for caring.

Prosocial and antisocial leadership styles can set the competitive or cooperative style for the family, team, group, or even nation. This seems as true for baboons as it is for humans (Sapolsky and Share, 2004). This may be because different styles and strategies of competitive behavior will try to create the social conditions and states of mind in those interacted with, that facilitate that competitive style and strategy. For example, in families, small and medium size groups, maintaining dominance and power by aggression and intimidation will need to stimulate fearful submissive states of mind, rather than open aggressive counter attacks in those around them. Prosocial leadership styles, on the other hand, will try to create states of mind, where others voluntarily and willingly follow and provide support and share resources (Barkow, 1980, 1989; Gilbert et al., 1995; Nesse, 2007).

**Figure 3:**
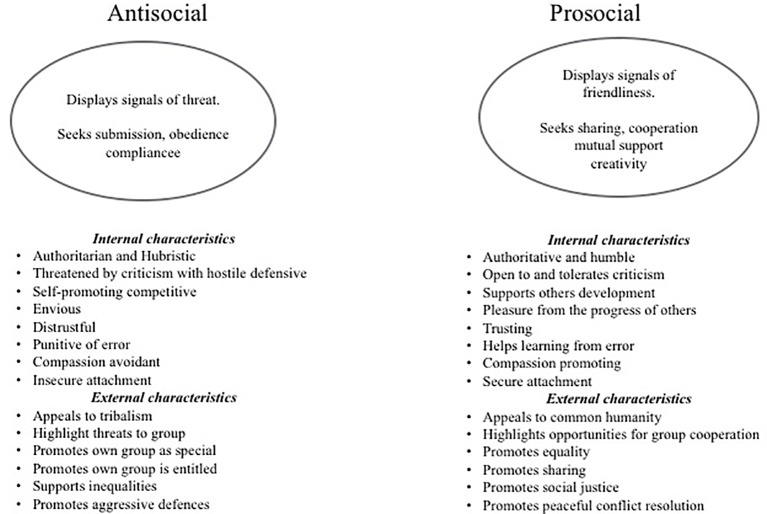
Rough overview of a selection of differential characteristics of antisocial and prosocial competitive and leadership styles.

Another model for competitive behavior and styles of leadership, that is also rooted in evolutionary models, is from the work of Zuroff et al. (2010) and Kelly et al. (2011). They identified three forms of leadership style that they labeled: dominant leadership, coalition building, and ruthless self-advancement. They based the dominant leadership items on dispositions to be dominant, assertive, and self-promoting to attain a leadership role. Coalition-building items were based on building cooperative coalitions, consulting with others, and seeking to compromise. Ruthless self-advancement was based on advancing self-interests by any means, including those that may be unethical, deceptive, and disloyal. Their work further indicates the existence of these two general categories of antisocial and prosocial strategies but with finer distinctions. Again, we see these styles as relevant to many forms of relationship building, e.g., parent, teacher, work manager because they are styles of social influence that ultimately are linked to styles of social competition and control. Bringing these themes together, we can depict the two dimensions of leadership in Figure 3.

Exploring how leadership styles represent patterns of social mentality activation and reciprocal interpersonal dances between interacting individuals means that competitive behavior cannot be analyzed simply at the level of an individual. Rather, analysis needs to be related to how different evolved algorithms, conscious and non-conscious, operate through the minds of individuals in communities. In a way then, we have come full circle from our earlier discussion of gene selection. Here we are considering how competition between *evolved algorithms and strategies* for competitive and reproductive behavior gets played out in the interactions between minds. What is particularly interesting is whether or not recent changes in human social contexts have also changed the arenas for the competition between these basic *evolved algorithms*. Agriculture maybe an example of a major ecological change that altered the competition of algorithms within populations.

## Social Contexts

One of the most fundamental questions in evolutionary psychology is the degree to which strategic plasticity is linked to processes such as epigenetics (Cowan et al., 2016; Conway and Slavich, 2017) and neuroplasticity (May, 2011). We have little data on how recently emerged social contexts have changed human genetic expression that can be passed through the generations. It is clear that different cultures activate different motives, strategies and behaviors. People behave in wars in ways they might never have envisioned possible in peace. Although slavery still exists, there are now international laws against what was previously endemic to human history. Gradually efforts are being made to address tribalism, particularly in the context of racism. Cohen (2001) shows how the distribution of resources, and the threatening nature of the social environments in which people live, have very major impacts on their attitudes and values, especially on whether they develop supportive and trusting or cheating and exploiting relationships with each other. Murder and crime rates vary greatly according to social context. People are not consciously choosing their strategies to be trusting or not, sitting down at night working out their strategies for the next day, but are operating with non-conscious algorithms rules and strategies. In his famous study of masculine identity, Gilmore (1990) showed that whether males present themselves as tough and fearless, or as gentle and peaceful, is highly related to the ecological and social context in which they mature.

Leadership styles not only reflect their social niche (e.g., different types of leaders are sought out in times of security versus times of threat) but can also shape it by promoting and stimulating different algorithms and motives within populations (Bass and Avolio, 1997; Ewest, 2017). Crucial are leaders who recognize the need to be very mindful of and contain the dark side of humanity as opposed to those who purposely stimulate it for their own self and group interests. Understanding the complex relationship between context, personality, and leadership style is central to understanding patterns of competitive behavior as they are played out in leadership roles in different types of relationship, organizations, and societies (Judge et al., 2009; Gilbert, 2018). Sapolsky (2017) gives an excellent review on a whole range of studies that indicate how powerful context, shared discourses, and systems of meaning are in shaping competitive and other behaviors. For example, he reviews studies that show that testosterone is typically associated with elevated competitive behavior and status seeking in males, but whether that status seeking is prosocial and altruistic or antisocial and potentially threatening and harmful is dependent upon the context in which status is being sought.

## Agriculture, the Emergence of Accumulators, and the Rise of the Antisocial Leader

There is general agreement that the advent of agriculture profoundly changed the context in which the epigenetic potential and neurophysiological architectures of the human mind played out its various strategies of mutual support and cooperation versus self- and kin-focused competition (Smith, 2002; Li et al., 2018). Agriculture supported the rapid expansion of food supplies and thereby group size, which in turn intensified competition and resource control and supported new forms of hierarchical social structures. The link between these processes is complex, with both ecological and social variables influencing the forms these hierarchical structures took and still take (Sheehan et al., 2018). While agriculture created many opportunities for the development of culture, freedom from famine, science, and medicine, history shows it has come at a terrible cost because it also facilitated the creation of social environments for intense and aggressive competitive behavior. Most of the major civilizations have come with extraordinary hierarchies of power, wealth, and where those in power have often used violence, threat, and torture to suppress disobedience to their rule and resource accumulation. In addition, the work of Keltner and colleagues has shown repeatedly that as people gain more power, they tend to become less compassionate and less interested in the wellbeing of those below them (Keltner et al., 2003; Keltner, 2016).

Importantly, agriculture created new means to wealth and trade, which enabled wealth and privilege to accumulate in kin groups to such extent that the difference between the haves, have nots, and have lots is now staggering (Piketty and Ganser, 2014). Clearly, human competition does allow huge discrepancy between winners and losers. However, as noted, this is partly due to the abnormal social environments we are now living in. In small hunter gatherer groups, such accumulations would have been severely sanctioned and indeed prestige was gained through sharing and altruism (Barkow, 1989; Boehm 1999). In modern societies we have created the exact opposite, where the wealthy have created political contexts to enable, even admire, and gain prestige from wealth accumulation and non-sharing. We have turned our natural regulators for the growth of resource disparates on their head.

Exploring the transition of humanity, from small hunter gatherer groups into the mega groups of today, that were facilitated by agriculture, can benefit the understanding of modern forms of competitive behavior and leadership (Mann, 1986). Crucial to the analysis of the emergence of strategies for resource accumulation and competitive leadership is the recognition of how different modern environments are from those of hunter gatherer groups of 10,000 years ago. This is known as the evolutionary mismatch hypothesis (Smith, 2002; Li et al., 2018). Well-known examples of problematic mismatches are modern diets and the ready availability of high fat, high salt, high sugar foods leading to problems of obesity and cancer (Smith, 2002). Another mismatch is in the provision of attachment and childcare with an overreliance on individual parents isolated in homes trapping children with potentially dysfunctional parenting. This stands in dramatic and tragic contrast to the environmentally open and multiple forms of care provision from a range of relatives (Hrdy, 2011; Narvaez, 2017). The manufacture and now the ready availability of drugs and alcohol, modern diet, and food availability, the sedentary lifestyles of sitting in front of computers and TV screens, the entrapment of women in marriages who are segregated from relatives and living in small homes which allows abuse, are but a few examples. There are a range of mental health problems, not to mention forms of criminality, that are clearly associated with modern environments (WHO, 2015).

The problems we have with modern competitive behavior and styles of leadership can also be partly linked to evolutionary mismatches. For example, in early hunter gatherer environments, people’s survival and reproductive success depended on social success, on cooperation with a variety of inhibitors on aggressive and wealth accumulation (Boehm, 1999). Ever since the advent of agriculture (Black et al., 2017) and the shift out of nomadic hunter gatherer groups, these balances have changed. Humans have had to contend with the creation of surplus, rapidly expanding group sizes and the formation of complex power hierarchies. They have also had to contend with inner clichés and styles of leadership that seek to control surplus, distribution, and ownership for self and kin. Outside of food availability, technological and medical advances, this transition has not always been conducive to human wellbeing. Historically, many humans have lived in abject poverty serving a small wealthy elite (Mann, 1986). As Galbraith (1987) highlighted, in modern industrial contexts the pursuit of wealth and resources is partly to escape the entrapments, limitations, drudgery, and misery of poverty. Poverty in a hunter gatherer, small mutually supportive, and free ranging group is very different to being entrapped in industrial cities, in cramped conditions of limited opportunity, impoverished social support, and relationships.

The human mind was suddenly confronted with a social ecology it was not adapted for and the consequences in many ways have been dire. In hunter gatherer societies, the striving for personal wealth and social control were limited by the social context and ecologies and the need to foster good relationships and reputations with each other (Boehm, 1999). Group size was small enough (100–150) for most people to know each other, reputations especially for helpfulness were important, and potentially reciprocal opportunities constantly possible (Dunbar, 2010, 2017). Once resources are potentially unlimited and group size increases, these social dynamics start to break down, and there is no natural constraint on wanting more and more, and personal ownership gains advantage over sharing (Mann, 1986; Keltner, 2016). Indeed, there is increasing evidence that as wealth increases so does the desire for more, and along with it, the advantage of accumulating, holding, and controlling rather than sharing. This is accompanied by the fear of loss of resources (stealing) and the emergence of laws to protect the property and wealth of the power elites. Historically, poor people who poached or stole from the wealthy could be hung (Galbraith, 1987; Van Kleef et al., 2008). Gaining dominance and power often goes with reducing empathic concern for those less fortunate or lower in the status hierarchy, not increasing it (Keltner et al., 2003; Keltner, 2016). The point is that some of the drivers for antisocial strategies and behaviors are new contexts of large groups (of non-reciprocating strangers) and opportunities for control over vast resources.

Expanding group size has other problems too. In many primate species when groups and troops interact, there can be violence between them. Jane Goodall reported how a group of common chimpanzees became big and then split into two groups, with the larger group subsequently hunting down and killing the smaller group. This became known as the chimpanzee wars (Goodall, 1990). In humans too, the orchestration of tribal violence is legendary, often fueled by aggressive leaders with social dominance orientation attributes and capable of stimulating hatred of the outsider (Gay, 1993; Ho et al., 2015). However, humans take it to completely different levels, with organized systems of training young males for one reason only, which is to fight, often being killed and maimed themselves for doing the same to mostly other young males in other groups and tribes. The enthusiasm by which young males can adopt these roles and commit the atrocities they do is a mark of the serious lack of human capacity to use compassionate, ethical and rational thinking to regulate destructive behavior and the dark side of humanity. Hostile tribal conflicts also reveals political and leadership failures to resolve disputes. Tragically, this is sometimes because it’s in the interests of leaders and their popularity back home not to have them resolved. In addition, many countries make huge profits from arms sales, that some leaders eagerly promote. Taking a moral view on how to work with strangers or outgroup members is tricky, because the evolution of moral thinking was linked to “in group” relating and for those most likely to reciprocate (Krebs, 2008). Given our primate heritage, it is easy to see why we have a range of antisocial innate dispositions that can be very easily stimulated by leaders in certain contexts.

For these and other reasons, the last 5,000 years or so are littered with dark triad leaders whose aggressive, expansive, competitive strategies for gaining dominance, often associated with sexual access and excess, have caused serious suffering to humanity. This has taken the form of brutal wars, genocides, slavery, the use of torture, extreme punishment as a form of threat and control, sexual exploitation, not to say the horrendous living conditions that impoverishment in towns has meant (Gay, 1993; Taylor, 2009; Plante, 2015). Indeed, in all forms of social organization, from families to teams, small groups, organizations, and even nations, aggressive male strategies in numerous contexts can exert very destructive influences on the minds of others (Gay, 1993; Lindholm, 1993; Glover, 2012; Schyns and Schilling, 2013). This is partly because leadership entails the ability to influence the attention, values, thinking, and emotions of subordinates and followers, including what frightens them and inspires them for good or for bad (Yukl, 2013). Green et al. (1998) showed that economic conditions can create vulnerabilities to hate crimes, but it is only when aggressive individuals set themselves up as leaders and orchestrate violent behavior that they become manifested in the community.

Not only have many historical leaders been very destructive, have advanced wars, tortures, and tribal violence, but they have been able to manipulate groups of supporters and subordinates close to them, who will carry out their threats and dictates (Kelman and Hamilton, 1989; Ignatieff, 1999). These are the henchmen and women. These are the secret police, various armies, and so on. Indeed, as we look back in history be it the Assyrians, Egyptians, Romans, Genghis Khan and the Mongols, the Indian Moguls, Chinese emperors, various popes, Napoleon, Hitler, Stalin, the vast majority of criminal gangs and on through to the modern day, the way “supporters” maintain the power base of aggressive (mostly) males, be it out of fear or admiration, is a serious problem for humanity. With certain kinds of leadership, it is very easy to get people to do cruel and immoral things (Zimbardo, 2006). Even today, many violent dictators and tyrants use their police and armies to subject their populations to horrendous violence to suppress dissent or rebellion.

Tragically, the world is awash with various subgroups with antisocial leaders who promote antisocial and harmful behavior and at times intense violence. This can be seen in various criminal gangs that set out to exploit people, hack computers, and create viruses, as well as the drug wars and murder rates of various countries and cities, sex trafficking, and the incitement of religious violence in many places around the world. At the center of these groups are often dominant antisocial leader males who try to inspire or intimidate those around them and hook into or harness these underlying motivations and algorithms that sit in the human mind, facilitating callous exploitation of others.

We should also note that in a world of not only increasing integration, but also increasing tensions and conflicts, it is in the competitive self-interest of some leaders with particular competitive styles to promote segregation “of the tribes and nations” rather than integration. Indeed, some styles of leadership can be hostile to external regulation. For example, some religious groups are resistant to moral dictates from outside or by governments. Some countries do not facilitate the working of (say) the United Nations and may pull out of efforts to bring more united legal systems into the world, such as through international courts, as well as international problems like climate change.

Although some species can appear to enjoy creating suffering, for example, killer whales playing with seals before they kill them, it is unclear if this is for conscious entertainment as such. Goodall (1990) suggests that although chimpanzees can be cruel, they do not really have an insight into the suffering they are causing. Humans, with their new competencies, clearly do and cruelty can be driven for entertainment (e.g., the Roman games). Underlying, evolved motivating systems are never far away. Hence, entertainment too is awash with mostly male competitive violence. Typically, the narrative depicts aggressive and morally lacking (outgroup) villains who do bad things to one’s own group, rape the women and kill the children, which then allows the good guys to come in with their own degrees of vengeful and at time sadistic violence. This creates excitement for audiences, and everyone goes home happy that the bad guys have got their comeuppance. Males are demonstrating their bravery and protective functions to their audiences. Audiences are cheering them on because they want to have demonstrations of who can be trusted and who is courageous enough and aggressive enough to protect them. Few will recognize the acting out of underlining algorithms that had been evolving over millions of years. Be it through violent video games focused on aggressive competitive behavior, or fascination with vengeful violence as story plots, the use of violence as a competitive strategy for gaining and defending resources is well homed in modern human societies.

Against this background of the potential gains from resource accumulation and even hostile forms of leadership, stimulating evolved motives and algorithms for compassionate and sharing behaviors, especially across ethnic and cultural groups, is difficult (Loewenstein and Small, 2007). Research is beginning to explore how to stimulate and promote courageous styles of leadership that are rooted in evolved motivational systems for prosociality (Hannah et al., 2011; Ewest, 2017; Zimbardo, 2018). Many commentators recognize that we need compassionate and prosocial ways of competing and sharing resources, which require leadership styles to work against tribal self-interest and tribal self-regulation, especially when it is harmful to the common cause of humanity. Basically, we need to create contexts where different motivations and algorithms that organize our mind for prosocial behavior can be stimulated. Relying on surface systems, such as beliefs or values, without addressing underlying evolved motivational systems that may well be operating unconsciously, will be limited.

## Life History and the Competitive Strategies

Finally, we draw attention to an area of research we think will play an increasingly important role in research on all kinds of human behavior, which is the link between contexts and genetic expression. This is especially important when we shift the focus from competing individuals to competing underlying algorithms and motivational systems. It is useful to keep in mind that “individuals” have a rapid turnover and do not survive for long, only the information in their gene-algorithms is passed from generation to generation. However, which motives and algorithms get activated and then become choreographed into a sense of self is very contextually related. This makes the contexts in which children grow and mature central to the kinds of minds we have and the algorithms we pursue (Cowan et al., 2016; Narvaez, 2017; Seppälä et al., 2017). Social contexts, from the day of conception, such as stresses and dietary factors affecting their mother, all the way through to the care and attention they received growing up (not only from the mothers and families, but in their local communities) will choreograph strategies and motives (Cohen, 2001; Narvaez, 2017). There is considerable evidence that the degree to which we are relatively prosocial or more callous in our competitive behavior is linked to early and current attachment styles and in particular the degree of security that individuals feel (Mikulincer and Shaver, 2007; Sheskin et al., 2014; Cowan et al., 2016; Seppälä et al., 2017). Individuals who grow up in relatively competitive or threatening environments become sensitized to the need to be self-focused, self-protective, and competitive (Sheskin et al., 2014). Indeed, Zuroff et al. (2010) found that ruthless self-advancement leadership styles were linked to avoidant attachment.

Evolution theories have highlighted the fact that human phenotypes have some degree of plasticity to them. This is partly linked to epigenetics and the fact that life experiences particularly, early life experiences, can alter the way genes are expressed (Shonkoff et al., 2012; Cowan et al., 2016). In addition, the impact of environments on different life strategies has been explored in what is called life history approaches (for a review, see Ellis and Del Giudice, 2014; Sheskin et al., 2014; Del Giudice et al., 2015). Environments that are relatively unstable with high levels of threat, social strategies, and phenotypes develop to orientate individuals to be relatively threat and self-focused, less cooperative, and more impulsive. These are called “fast” life strategies because individuals tend to come into reproduction earlier and are less investing in their primary relationships. By contrast, in stable, safe, and cooperative environments, survival and reproductive strategies are more advantaged by sharing and altruistic behavior. These are called “slow” life strategies.

Fast strategies involve more risky engagement with life, power seeking, with potentially high gains of accumulating resources to self and lower interest in investing or caring for others. By contrast, slow strategies are more common to stable and safe environments and sharing (Ellis and Del Giudice, 2014). Importantly too, it may not be that early environments are potentially threatening in terms of being abusive, but they can be neglectful. A lack of parental warmth may be especially linked to the maturation of unemotional-callous traits (Henry et al., 2018). These rearing experiences leave children with an overly developed sense of having to be highly self-reliant. Associated with these difficulties, such as callousness, are ones that may be linked to difficulties in processing their own (difficult) emotions. For example, Shirtcliff et al. (2009) suggest that they may be alexithymic to their own emotions, and indeed, they offer some neurophysiological evidence to support this. These authors suggest that the callousness to other people’s suffering is partly linked to an inability to process their own emotions, and therefore, mirror neurons and theory of mind systems do not work well for them. In essence then, early life experiences may orientate individuals to be competitive in different ways. Be it in leadership roles, or in general, research is increasingly focused on these kinds of interactions and creating contexts that have the best chance of promoting prosocial behavior in ourselves, in our relationships, organizations, and politics.

## Conclusion

This article has explored the nature of human competitive psychology and leadership as emerging out of pre-human motives and algorithms for competitive behavior. The central theme of the article is that we have the potential for different types of competitive behavior along dimensions of antisocial and prosocial. These dimensions are reflected in many styles of relating, but especially in leader-follower and dominant-subordinate relations.

We have highlighted that while aggressive forms of competing and seeking to control others are still endemic in human relating, humans also have a need for approval, acceptance, and being connected to supportive communities. Indeed, these are basic human needs that orientate us to mental and physical wellbeing (Gilbert, 2009, 2018; Cowan et al., 2016; Narvaez, 2017; Seppälä et al., 2017). The reason for highlighting the evolutionary underpinnings of competitive behavior and leadership is because without an understanding of our innate motivational systems and their algorithms, and the contexts that bring them to life, we may struggle to create the styles of leadership and the social contexts which support wellbeing, social justice, and fairness. Models of leadership that simply articulate different behavioral styles or create wish lists for how leaders should be, but without recognition of the powerful conscious and unconscious motivational systems that guide human behavior, may falter.

Time and time again, be it in industry or in politics, antisocial leaders can be very damaging even if they appear confident, competent, and appeal to tribal self-interest (Lipman-Blumen, 2005). What is now required are models of leadership that help to articulate much more clearly prosocial and antisocial forms of leadership, identify individuals who lack prosocial competencies and motives (even if they can fake them), and contribute to an understanding of how to counteract some of the evolved algorithms that drive the dark side of humanity. We need to especially improve our science of understanding how and why communities gravitate to antisocial leaders and how to address this deep problem for humanity (Lipman-Blumen, 2005; Cheng et al., 2014). Simply put, we cannot afford to continue to endorse antisocial leaders. We urgently need to develop our science of leadership that helps us understand how to counteract their appeal and support those with prosocial interests.

## Author Contributions

PG and JB were involved in all aspects.

### Conflict of Interest Statement

The authors declare that the research was conducted in the absence of any commercial or financial relationships that could be construed as a potential conflict of interest.
